# Different level of virtualization of sight and touch produces the uncanny valley of avatar’s hand embodiment

**DOI:** 10.1038/s41598-019-55478-z

**Published:** 2019-12-13

**Authors:** M. D’Alonzo, A. Mioli, D. Formica, L. Vollero, G. Di Pino

**Affiliations:** Neurophysiology and Neuroengineering of Human-Technology Interaction Research Unit, Campus Bio-Medico University, via Alvaro del Portillo, 5, 00128 Rome, Italy

**Keywords:** Consciousness, Social behaviour

## Abstract

Humans increasingly often act through virtual and robotic avatars, which can feed back to their user only virtual sensory information. Since avatar is user’s embodiment and body image is mostly based on senses, how virtualization of sensory inputs affects avatar self-attribution is a key question for understanding nowadays human behavior. By manipulating visual and tactile inputs in a series of experiments fashioned after the rubber hand illusion, we assessed the relative weight of the virtualization of sight (*Real*, *Robotic*, *Virtual)* and of touch (*Real*, *Virtual*) on artificial hand embodiment. Virtualization decreased embodiment, but unexpectedly lowest embodiment was found when only one sense was virtual. Discordant levels of virtualization of sight and touch elicited revulsion, extending the concept of the uncanny valley to avatar embodiment. Besides timing, spatial constraints and realism of feedback, a matched degree of virtualization of seen and felt stimuli is a further constraint in building the representation of the body.

## Introduction

One of the most astonishing behavioral revolution of present everyday life, based on deep technological and social changes, is that humans are increasingly often acting through virtual or robotic substitutes of their physical body.

On one side, today’s robots are widespread in our everyday life; they are increasingly used for tele-operated activities, domestic tasks, for companionship and entertainment. Social robots, designed to actively interact with humans also in the emotional domain, have often hands and faces resembling the human body^1,2^.

On the other side, virtual human-like-avatars have begun to be employed as proxy of individuals to substitute their real human body and facilitate social and environmental interaction in an immersive virtual reality setting for gaming, social network and entertainment. Soon, human-like avatars will be employed in other on-line activities such as e-travel, e-commerce, banking etc.

For centuries, humans built social relations and behaved in the environment by physically taking advantage of their body; as side effect, telecommunications, internet, social networks, and remotely controlled robots can deprive human behaviours of the human body. In exchange of easiness of action, interactions became less experienced and depersonalization makes people feel less responsible of their actions.

In any case, this behavioural revolution opens novel fascinating neuroscientific questions. Since avatars can feed back to their users only virtual or substitutive sensory information, (1) how the virtualization of senses affects how much the user perceives the avatar more real and human-like? (2) How such realism can be evaluated?

1. Indeed, the interaction through a teleoperated robot, which is real but transposed (i.e. coming from another place), and the interaction through a virtual avatar in a virtual environment can be coded to their users only through substitutive sensory feedback. Today, available technology allows to relay such information only with virtual, mostly cartoonish, visual feedback and modality-mismatched haptic feedback where vibration is delivered instead of touch. Virtualization of sensory feedback is likely to decrease the realism of the avatar.
2. Hitherto, the realism of virtual and robotic avatars has been evaluated through the acceptance of human subjects interacting with them, mainly through questionnaires^3,4^. Anthropomorphism of the appearance^5^, of movements^6,7^ and behavior^8^, and the ability to provide a close-to-natural physical interaction with people^1,9,10^, seem to determine the acceptance of humanoid robots and avatars.

However, an avatar is defined as an incarnation of a human form in an artificial or virtual image, hence the self-attribution of the avatar or part of it, would most likely be a more direct proxy of its realism.

Neural correlates of self-attribution depend on multi-sensory perception^11–13^; e.g. the attribution of a limb to the self depends on the integration of the afferent somatic stimuli and visual feedback from the limb. Thus, it’s likely that the more the sensory inputs from the avatar resemble physiologic sensory feedback, the more its embodiment will be effective. The low-fidelity and modality-mismatch of visual and somatosensory feedback coming from an avatar are features that could make the sensory code less physiologic.

Considering that many applications of robots for teleoperated activities and virtual avatars use especially the hands, and taking advantage of the huge body of literature on the embodiment of fake hands, we chose to employ the rubber hand illusion (RHI) paradigm to estimate individuals’ self-attribution of the avatar. This paradigm tests the depth of a body ownership illusion, i.e. experiencing a rubber hand to be part of the own body, that emerges when a visible fake rubber hand and the subject’s hidden hand are sincronously stroked with paintbrushes^11^.

VR and haptic technologies can be employed to easily manipulate the perceived scenario in a virtual version of the typical RHI paradigm, eliciting the so called Virtual Hand Illusion (VHI). One of the first experimental versions of VHI was performed by stroking a 2D video image of a hand projected on a table^14^. Subsequently, with the progressive improvement of this technology, the systems for virtual reality have become more immersive and realistic. Several studies employed a projection screen placed in front to the user associated with head-tracked stereo-lenses in order to reproduce the objects projected on the screen from the participant’s point of view and determine the virtual image depending on the user’s head direction^15–18^. More recent works employed head-mounted displays (HMD) showing real-time stereoscopic video imagery^19–24^. Vibrotactile devices are the most used to reproduce the contact and the interaction with objects within the virtual environment in VHI paradigm^15,20,22^ because they are usually little, inexpensive, power-efficient and can be easily fitted on clothes worn by the users. In the past, by employing VR headset in virtual environment and substituting the touch of the paintbrush with vibrators, it has been shown that the illusion survives to the virtualization of this sensory input.

In the past, by employing VR headset in virtual environment^21^ and substituting the touch of the paintbrush with vibrators^25^, it has been shown that the illusion survives to the virtualization of this sensory input. To date, no studies directly compared the depth of the illusion induced by the typical RHI paradigm with the same paradigm in a virtual environment by taking in consideration both visual and tactile sensory input.

This study assessed the relative weight of progressively virtualizing visual and tactile sensory inputs in the induction of the embodiment of the hand. Being virtual something that is such in the effects, though not formally in reality, with progressive virtualization it is meant decreasing the adherence to the physical reality, while trying to reproduce the information content of the sensory inflow. Sensory substitution has important applications in Prosthetics. Thus, identifying the impact on the VHI of the virtualization of sensory feedback may suggest the sensory features (appearance and characteristic of somatosensory stimulation), which are more likely to determine the self-attribution of prosthetic limbs.

**Figure 1:**
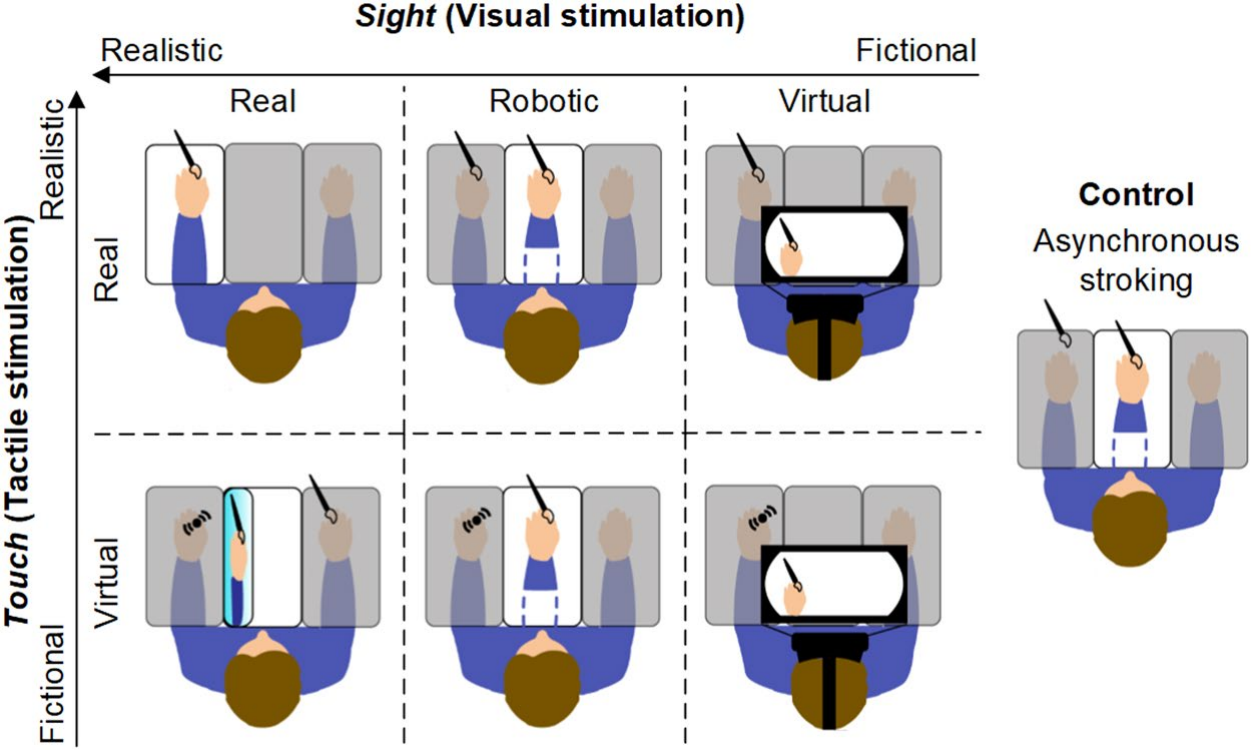
Schematic illustration of the six experimental conditions plus the control. Along the rows the degree of virtualization of visual stimulus increases: (Factor: *Sight*); along the coloumns the degree of virtualization of tactile stimulus increases: (Factor: *Touch*). In the right the control condition.

Three levels of virtualization of the visual sensory input (factor *Sight*: *Real*, *Robotic*, *Virtual*) and two levels of the virtualization of tactile sensory input (factor *Touch*: *Real*, *Virtual*) were employed to design a 3 × 2 matrix of six experimental conditions readapted from the RHI classic paradigm (Fig. 1).

Subjects saw a hand stimulated by a paintbrush and synchronously felt the stimulation on their hand. In *Sight-Real*, the seen hand was their real own, in *Sight-Robotic* it was a robotic hand with a rubber cover, and in *Sight-Virtual* a virtual hand was displayed though a Head Mount Display (HMD). Participants’ hand was stimulated with a real paintbrush in *Touch-Real*, and with vibrators in *Touch-Virtual*.

Additionally, an asynchronous condition (*Control*), where a small temporal delay between the stroking of the rubber hand and real hidden hand was introduced, was employed as no illusion condition. The strength of the illusion among the different conditions was quantified by a self-evaluation questionnaire and the proprioceptive drift. Three illusion outcomes were derived from the questionnaire: RHI index, vividness and prevalence (see Materials and Methods). The *Sight-Real_Touch-Real* condition was carried out to evaluate the full-scale value reachable by the employed measures of embodiment.

## Results

### Embodiment measures analysis

**Figure 2:**
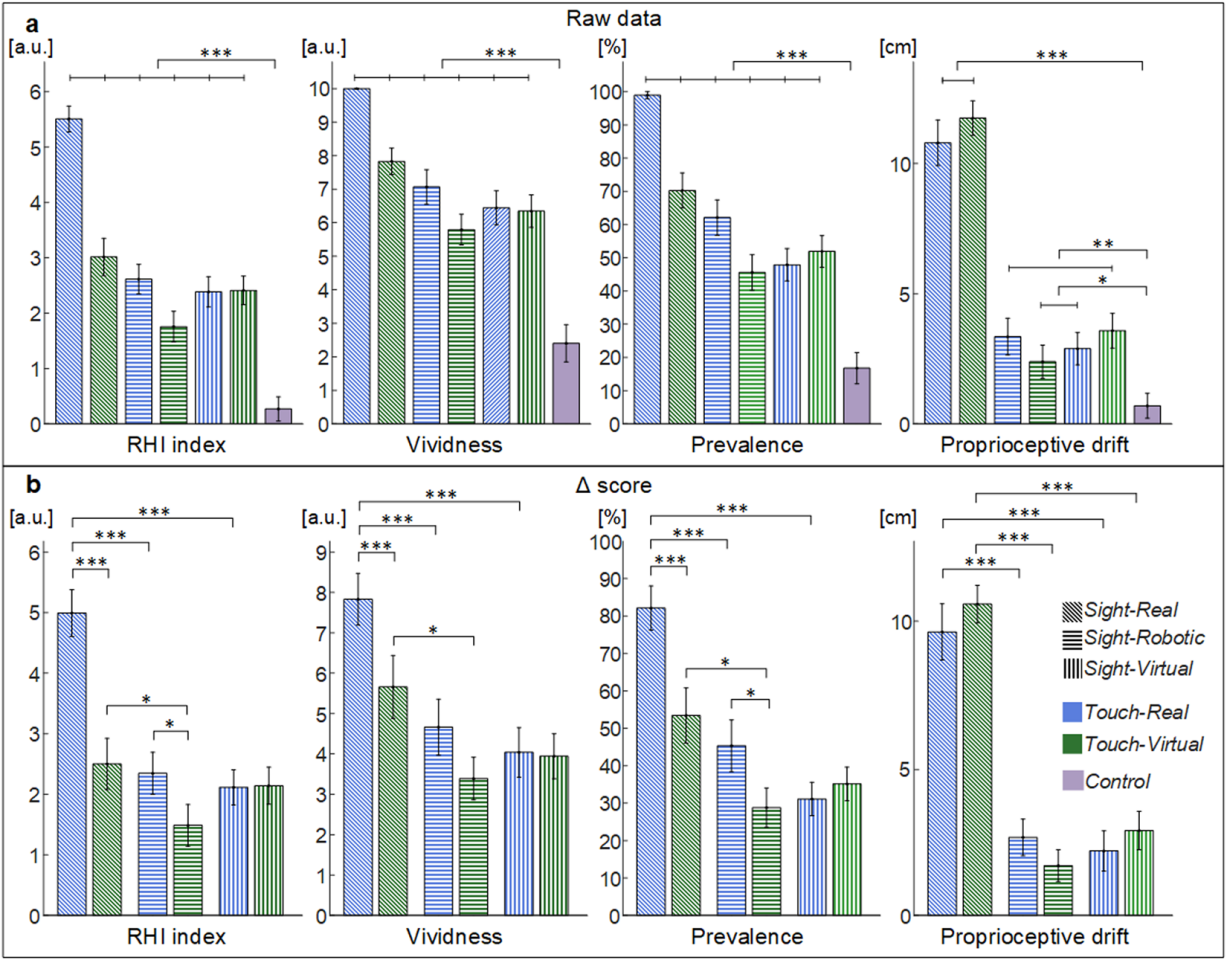
(**a**) Values (mean ± sem) of the RHI index (difference between the means of pooled illusion statements and control ones), vividness, prevalence rating and proprioceptive drift for different conditions. The horizontal lines indicate a statistical difference between a synchronous condition and the asynchronous one. (**b**) Δ scores (mean ± standard error) of the RHI index (difference between the means of pooled illusion statements and control ones), vividness, prevalence rating and proprioceptive drift for all the different conditions. Different fill patterns of the bars were employed to differentiate the degree of *Sight* virtualization, whereas different colors differentiate the degree of *Touch* virtualization. * indicates a p-value < 0.05; ** indicates a p-value < 0.01; *** indicates a p-value < 0.001.

The group of 26 tested subjects were found to be not-suggestible (questionnaire illusion statements mean rate vs questionnaire control statements mean rate: p < 0.001). The illusion arised in all six experimental conditions, since all the collected measures (i.e. *RHI index*, *vividness*, *prevalence* and *proprioceptive drift*) were significantly higher than in the control condition of typical asynchronous RHI stroking, as highlighted by the pre-planned pairwise comparisons (RHI index, vividness and prevalence: p < 0.001; proprioceptive drift: p < 0.05) (Fig. 2a).

Both the virtualization of visual and somatosensory feedback decreased all the collected measures of the embodiment of the hand. Considering each Δ score measure (difference between condition and control in each subject) independently, there were main effects of the virtualization of sight and of touch, and the effect of their interaction, for the outcome of the RHI index (*Sight:* F_2,136_ = 27.8, p < 0.001; *Touch:* F_1,136_ = 34.1, p < 0.001; interaction: F_2,136_ = 23.2, p < 0.001), vividness (*Sight:* F_2,136_ = 11.5, p < 0.001; *Touch:* F_1,136_ = 12.0, p < 0.01; interaction: F_2,136_ = 6.0, p < 0.01) and prevalence of the illusion (*Sight:* F_2,136_ = 18.2, p < 0.001; *Touch:* F_1,136_ = 15.0, p < 0.001; interaction: F_2,136_ = 10.8, p < 0.001); only the main effect of the virtualization of *Sight* was identified for the *proprioceptive drift* (*Sight:* F_2,136_ = 55.8, p < 0.001; *Touch:* F_1,136_ = 0.3, p = 0.58; interaction: F_2,136_ = 2.1, p = 0.07) (Fig. 2b).

### Principal component analysis

The results were very consistent among the four collected dependent measures^11,26^, so that we decided to merge those measures with a PCA algorithm for dimensionality reduction. The resulting first component (Principal Component of Embodiment: 1PCE), which explained 61% of the variance of all the four measures, was a single parameter comprehensive of self-reported and behavioural measures of the RHI^27^.

**Figure 3:**
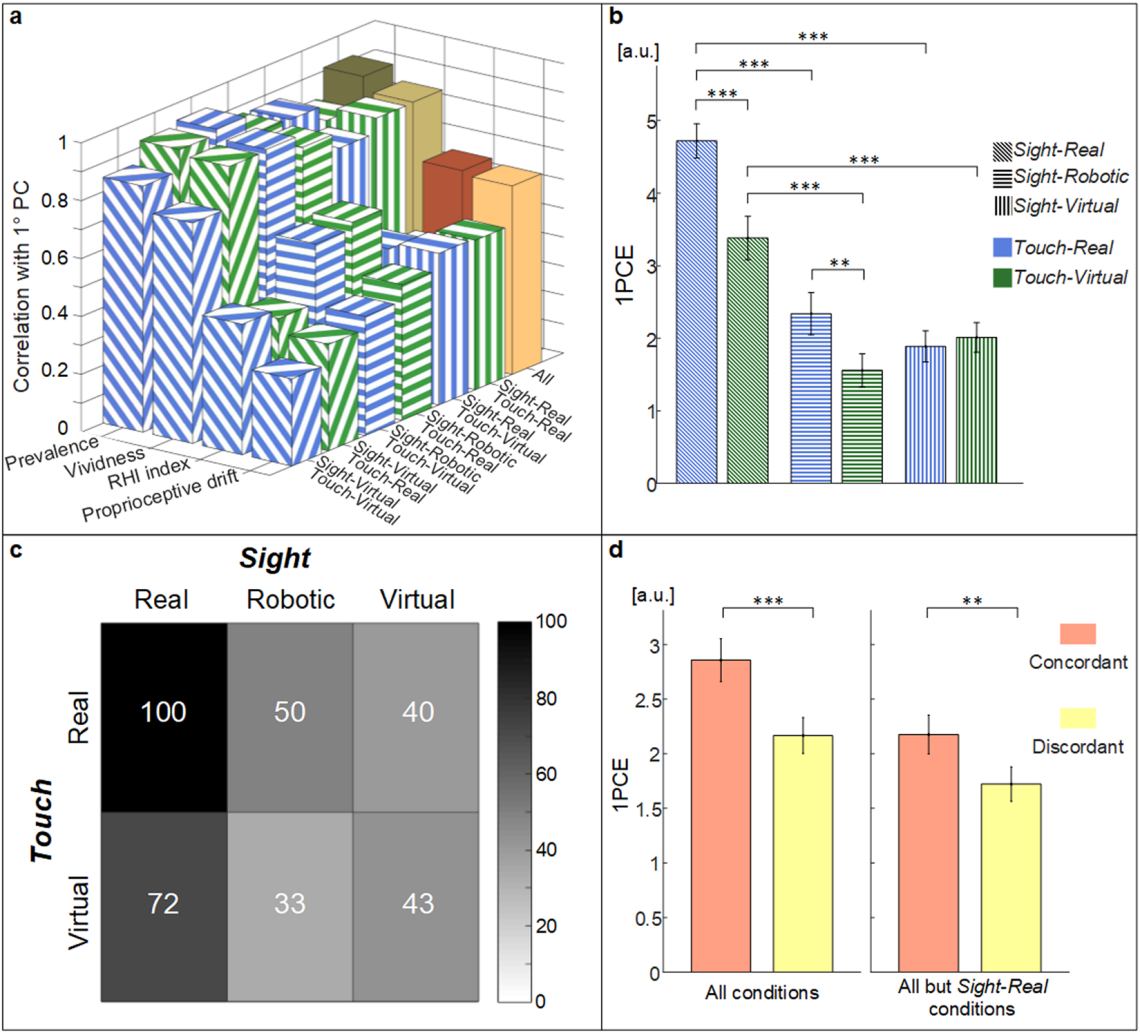
(**a**) Correlation coefficients between the four measures and the first component of the PCA calculated pooling all the conditions together (solid colored bars), and independently for each condition (fill patterned bars) (**b**) 1PCE (mean ± sem) for the different conditions. The horizontal lines indicate a statistical difference between conditions highlighted by the pre-planned pairwise comparisons. (**c**) Mean percentage of 1PCE for different conditions with respect to the standard embodiment of a limb (*Sight-Real_Touch-Real*). (**d**) 1PCE (mean ± sem) of the pooled data of concordant and discordant conditions. In the top panels (**a,b**) different fill patterns of the bars were employed to differentiate the degree of *Sight* virtualization, whereas different colors differentiate the degree of *Touch* virtualization. ** indicates a p-value < 0.01; *** indicates a p-value < 0.001.

The 1PCE was robust so that calculating it pooling all the conditions together and calculating it in each condition separately did not significantly change its correlations with the four collected measures (p > 0.05) (Fig. 3a) (see Supplementary Material).

The virtualization of both visual and somatosensory input significantly affected such parameter (*Sight:* F_2,136_ = 36.2, p < 0.001; *Touch:* F_1,136_ = 21.0, p < 0.001; interaction: F_2,136_ = 13.9, p < 0.001) (Fig. 3b).

Considering the 1PCE achieved in the condition of both real inputs (*Sight-Real_Touch-Real*) as full-scale value (i.e. 100%), embodiment decreased to 50% (*Sight-Robotic_Touch-Real*) and 40% (*Sight-Virtual_Touch-Real*) with the virtualization of visual input, and from 72% (*Sight-Real_Touch-Virtual*), to 33% (*Sight-Robotic_Touch-Virtual*) and 43% (*Sight-Virtual_Touch-Virtual*) with the virtualization of touch (Fig. 3c). All the tested conditions were significantly different from the condition of both real inputs (*Sight-Real_Touch-Real*) (pre-planned post-hoc analysis p < 0.001), and one from the other, except for the difference between *Sight-Virtual_Touch-Real* and the two contiguous conditions (*Sight-Robotic_Touch-Real* and *Sight-Virtual_Touch-Virtual*), whereas the difference between *Sight-Robotic_Touch-Virtual* and *Sight-Virtual_Touch-Virtual* did not overrun Bonferroni correction (Fig. 3b).

Surprisingly, the two main factors *Sight* and *Touch* showed a significant interaction (F_2,136_ = 13.9, p < 0.001), exhibiting a trend change; in particular, *Virtual-Touch* condition showed a significant decrease of the embodiment level in both *Sight-Robotic* and *Sight-Real* conditions; whereas in *Virtual-Sight* condition the difference between *Virtual-* and *Real-Touch* was not significant. Conversely, the mean value of embodiment when both inputs were virtual was higher than the two contiguous conditions. This suggested us to test whether the concordance between the level of virtualization of visual and the level of virtualization of somatosensory inputs had a significant impact on the avatar’s embodiment. Concordance of inputs had a strong main effect considering all conditions (p < 0.001), and even considering all but the *Sight-Real* conditions (p < 0.01), to exclude that the effect would have been due only to the high level of embodiment achieved in this condition (Fig. 3d).

It is worth noting that, considering only the *Sight-Robotic* and *-Virtual* conditions, there was not significant main effect of *Sight* and *Touch*, but only a strong interaction between them (F(1,25) = 9.7, p < 0.01). Similar findings were also obtained by applying such analysis to the typical illusion outcomes: there is a strong interaction for RHI index, vividness and prevalence scores (see Supplementary Materials). This means that the presence of a statistically significant interaction persists when considering all but the *Sight-Rea*l conditions. It suggests that the effect of the concordance in degree of virtualization is not due to the *Sight-Real* conditions.

**Figure 4:**
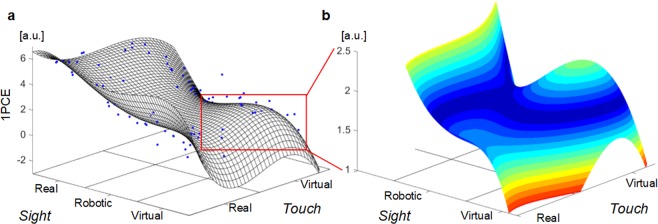
(**a**) Best fit surface (R^2^ = 0.80) of the number of points that represent each participant in each condition. The points were identified by three coordinates in a 3D space where z-value was the 1PCE, x-value was the marginal effect on the 1PCE of *Sight* and y-value was the marginal effect of *Touch*. The marginal values were scaled as x and y coordinates to fit their distribution within the 2D spatial square (the black lines on the x-y plane) of the relative condition. (**b**) Particular of the valley: the different colors indicate different level of 1PCE.

Thus, the decrease of embodiment (1PCE) was due to both virtualization of inputs and discordance between the level of their virtualization. In order to highlight this phenomenon beyond the effect averaged across subjects, each participant in each condition was represented as a point in a 3D space, being the dependent variable (z-value) the 1PCE and the other two coordinates the relative contribution of each single factor (*Sight* and *Touch*) on the measured effect. The points were fitted on a polynomial curve quartic in x coordinate and cubic in y one (R^2^ = 0.80). The fitted surface had valley shape with a decrement and a local minimum within the conditions *Sight-Robotic_Touch-Virtual* and *Sight-Virtual_Touch-Real* and an increment with a local maximum within the condition *Sight-Virtual_Touch-Virtual* (Fig. 4).

## Discussion

This study was designed to assess the relative weight of the virtualization of visual and somatosensory inputs in the embodiment of a robotic or virtual avatar, and in particular of the hand. This may have several possible applications, such as in gaming or in the development of future prosthetic hands which employ sensory substitution feedback.

We reckon that the embodiment is a more direct mean to assess the realness and human-likeness of an avatar, and of the easiness of interaction, than simple subjective acceptance of the avatar. Having knowledge of these relative weights is fundamental in order to design easily embodiable devices and applications in virtual environment and in robotics, and to better understand humans behaving through them.

The main hypothesis of the study was that the progressive virtualization of visual and tactile stimuli, along a scale that goes from completely real to completely virtual stimuli, would have decreased avatar hand embodiment.

This is the first time that embodiment in the RHI is directly compared with embodiment in the VHI and, moreover, the first time that they are compared to the standard embodiment of an owned limb (i.e. Real vision and consistent tactile feedback: *Sight-Real_Touch-Real* condition).

A certain level of embodiment was present in all the real, robotic and virtual condition, thus our results confirm that embodiment can be induced through sensory substitution and in a virtual environment. However, the depth of embodiment differed across conditions, suggesting that the use of the VHI as an alternative to the RHI should be done with caution.

As expected, all the participants reported higher values of the four collected measures in *Sight-Real_Touch-Real* compared to all the other conditions, justifying our attempt to use this condition to derive the full-scale value for the collected measures in our experimental setup and circumstances. Indeed, the impact of experimental setup and circumstances led some participants not to report the maximum rating in some illusion statements (S1 and S2) and in prevalence, because they did not immediately realize that the stroked visible hand was truly their own.

Despite the four collected measures are particularly sensible to different aspects of the embodiment (i.e. intensity of sensation for vividness, continuance of the sensation for prevalence, depth of the illusion with respect to the suggestibility of the subject for RHI index and spatial update of sense of hand’s position for proprioceptive drift), they manifested the same trend of change across all the conditions. Indeed, the correlation analysis between the four measures and the first component of the PCA calculated pooling all the conditions together, and independently for each condition was not different. This is in support of the robustness of the employed experimental design and of the use of the 1PCE as single derived parameter to describe the embodiment.

In the RHI, a tricky match between sight (i.e. the brushstroked rubber hand) and somatosensory feedback (i.e. perceiving the real hand brushed) co-exists with several possible mismatches, such as the one between the seen position of the rubber hand and the felt position of the real hand. The extent of the illusion depends upon how much the induced false match overwhelms the mismatches. In this study, different conditions corresponded to different modulation of the mismatch between seen and felt stimuli, so that the changes in embodiment may be interpreted in such framework.

However, our 3 × 2 experimental matrix was specifically designed to modulate the adherence of the sensory feedback to the physical reality, thus a more accurate framework of interpretation of our results is the one which links them to changes of virtualization of visual and somatosensory feedback, which was the main hypothesis of this work. The virtualization significantly decreased embodiment, both when the scores of the questionnaire, prevalence, vividness and proprioceptive drift were analysed separately or condensed in the 1PCE. Moreover, we found one unexpected result. The decrease of embodiment due to the virtualization of *Sight* and the one due to the virtualization of *Touch* are not independent one from the other, and they even seem to interact in a bimodal way, or at least their rate of decrease does. Specifically, the loss of embodiment due to the virtualization of *Sight* is tempered if also *Touch* is virtualized and *viceversa*. Concordance between the levels of virtualization of the two sensory pathway matters; indeed, this factor was found to be significant.

We attributed changes of embodiment to the progressively virtualization of visual and sensory inputs; however the assessment of virtualization we employed may have been affected by the fact that we did not assess a continuous scale of virtualization, but only six scenarios, and we cannot guarantee that they were separated by steps of equal magnitude.

Despite such limit, it can be speculated that the more a sensory modality is virtualized, the more the embodiment process is robust against virtualization of the other sensory modality, as if the subject does not notice it; in other words, the more a sensory input (e.g. the appearance of the environment and hand’s aspect) is close to reality the more a mismatch between the seen and felt tactile stimulation is perceived. A similar result was previously reported with the 2 dimensional/3 dimensional conflict: a 2D projected image of hand brushed by a real 3D paintbrush elicited a milder illusion than a projection of a 2D video of a brushed hand^14^.

A possible explanation of the reason why the virtualization of both sight and touch (*Sight- Virtual_Touch- Virtual*) seems to show a higher level of embodiment with respect to the discordant conditions, expecially the *Sight-Robotic_Touch-Virtual* one, may be that such condition is the one commonly employed in virtual applications, thus participant may be familiar with it. However, we think this unlikely because participants’ group was eterogeneous and more than half of them was naïve for immersive VR.

The bimodal trend linking virtualization of visual and somatosensory inputs with embodiment of virtual and robotic avatar hands is in line with the *uncanny valley* that has been described between appearance and subjective acceptance of robots^5^ and of virtual avatar^28^. Indeed, when we spread the mean of 1PCE in each condition into a multitude of points each one representing a participant, and we separated the contribution of the two factors (Virtualization of *Sight* on the x-axis and virtualization of *Touch* on the y-axis), the best fit surface clearly shows a region of depression of the effect (i.e. a valley), located within the discordant (*Sight-Robotic_Touch-Virtual* and *Sight-Virtual_Touch-Real)* conditions, surrounded by a region with higher effect.

The concept of the uncanny valley suggests that the relationship between the degree of an object’s resemblance to a human being and the emotional response to the object increases with the object’s human likeness, but when humanoid objects appear almost, but not exactly, like real human beings they elicit uncanny, or strangely familiar, feelings of revulsion in observers. Recently, the valley has been also shown for subjective experience in virtual reality when haptic feedback cannot be justified by what the subject sees on the display^29^.

Therefore, this work extends the concept of the uncanny valley beyond avatar’s appearance to cover the embodiment of avatars, and beyond a single sensory modality, either vision or touch, towards the concordant vs discordant interaction of the two in matching seen and felt stimuli to build the body image.

Self attribution arises from a coherent match of visual and somatosensory incoming information. Hitherto, big efforts have been spent to identify in the real world the constraints of such coherency, thus for a successful embodiment; time^30^, spatial^31^ and appearance^23,32^ constraints have to be satisfied to allow the illusion to arise. Going beyond the real world in a virtual or transposed scenario, our findings for the first time candidate the match between the degree of virtualization of seen and felt stimuli as a further constraint in the embodiment process.

It can be speculated that, in order to be immersed in a virtual visuo-haptic environment, our brain changes focus of input modality and begins to increase trustiness on both visual and tactile virtual sensory inputs, while reducing its trustiness in real feedbacks or perceiving as annoying the mismatch. Thus, contrary to what hypothesized when we planned the study, the amount of realism of a visuo-tactile sensory feedback is not an absolute value in building the representation of the body, and the coherent match between the degree of virtuality needs to be taken into account as a determinant factor. Coherent virtualization of different sensory pathways may be crucial also for developing easily embodiable devices and for re-personalizing depersonalized behaviours.

The valence of the results of our study may have been affected by some choices made in experimental procedure:

I. We included only a single common control condition based on asynchronous tactile stimulation of the rubber hand. This has been done to reduce the length of the experimental sessions and preventing the progressive lack of novelty.
II. We tested two conditions where the real hand was stimulated, which of course does not induce a proper perceptual illusion. Testing the real hand stimulation has been done in order to have a full scale measure of the reachable level of embodiment. To implement such conditions we had to delete three control items of the questionnaire and how we evaluated the proprioceptive drift. Moreover, when the touch was virtual we employed a mirror to show a fake brush-stroking while the real hand was instead stimulated by vibrators. It’s worth to note that the type of interaction between Sight and Touch factors, that is the main finding of our work, still persists if the *Sight- Real* conditions were not analyzed.
III. We employed currently-available VR system and vibrotactile stimulators. The virtualization of tactile stimulation achievable with vibrators lacks the richness of tactile details proper of brush-stroking, as well as the richness of graphic reproduction of real objects is still limited by the performance of the present technology. In the next future, the daily improving of 3D graphics rendering and haptics technology may be able to modify the richness of the virtual experience and our findings.

## Materials and Methods

### Test platform

The employed test platform was a flexible hardware/software system tailored for the rapid deployment of cognitive experiments. The platform was designed to simplify the development and management of virtual setups populated with cyber-physical objects and to manage and control their interaction with the user. The platform was composed of a Head Mounted Display (HMD) (HTC Vive), an array of vibrotactile stimulation devices (Precision Microdrives Inc.) controlled by a PIC development kit (CCS Inc.), a VR software application (Unity3D) and an ad hoc physical setup where the participant was seated.

The VR application showed a scenario in first person perspective through the HMD where the participant saw a brushstroke stimulation on a virtual hand (Fig. 1). In such environment, participants were head-tracked to show the virtual scene according to the head position and orientation. The position of the hands could be tracked by using the controllers associated to the HMD.

The VR application was capable of providing a visual and auditory stimulation to the experimenter in order to synchronize his movements to the events of contact and release of the virtual paintbrush (i.e. a metronome). Additionally, it was possible to send a series of commands by serial communication to the PIC board which controls the vibrotactile devices (two for the specific experiments); in this way the vibrotactile devices could provide a tactile stimulation on the participant’s real hand with different timings with respect to the virtual brushstroke. The vibrators could be also sequentially activated by the experimenter by using a push-button connected to the PIC board. Each vibrator could be activated to operate at a pre-defined vibration frequency (165 Hz) or deactivated.

During the experiments, the participant sat up in front of an ad hoc built experimental setup. Such structure was composed of a table (dimensions [Length × Width × Height]: 120 × 60 × 70 cm) split into three compartments (dimensions: 40 × 60 × 21 cm) by two dividers. A two-way mirror was placed on top to cover the compartments. Each compartment had its own illumination system granted by a strip of LED lights, so the content of each compartment could be visible to the participant only if the experimenter turned on the relative strip of LEDs by an electrical switch. The dividers between the compartments could be removed and substituted by a mirror of the same size. Such frame was employed to perform the experimental conditions in real environment.

### Participants

After signing a written informed consent including the permission for their images threatment, 26 volunteers (12 females, age = 29 ± 3 [mean ± standard error]) naïve to the RHI participated in the study. All participants were healthy and verbally reported to have normal hand sensation and normal, or corrected to normal, vision. Three of them were left-handed. Experiments were conducted according to the Declaration of Helsinki and after approval of the Ethics Committee of Università Campus Bio-Medico di Roma (EMBODY protocol).

### Experimental procedure

All the experimental conditions were performed in a within-subject randomized-order design. The rows (*Sight*) and the columns (*Touch*) of the matrix in Fig. 1 represent the two independent variables of the study, and each cell of the matrix corresponds to an experimental condition: *Sight-Real_Touch-Real, Sight-Robotic_Touch-Real, Sight-Virtual_Touch-Real, Sight-Real_Touch-Virtual, Sight-Robotic_Touch- Virtual, Sight-Virtual_Touch- Virtual*.

In all conditions, participants comfortably sat on a chair in front of the experimental platform, with forearms placed inside the two compartments, palm-down oriented. Tactile stimulation was delivered at a frequency of about 1 Hz and the duration was of 0.6–0.7 s. In each condition, the experiment lasted 90 s.

The classical asynchronous RHI procedure was employed as a supplementary (seventh) control condition. It has been selected as it is the one usually employed as standard no-embodiment condition in real environment and it was used as the “lowest common denominator” to remove the baseline embodiment offset related to each participant in each condition, and to compare all the conditions among them. Moreover, it has been chosen to employ a single control in order to reduce the overall number of repetitions of the experimental paradigms. After switching the light on, the experimenter started to stroke with two identical paintbrushes the dorsal surface of the index finger of both the visible fake hand and of the hidden real hand. The fake hand was a left rubber hand matching the participant’s gender and placed in the central compartment of the structure at a distance of 15 cm from the left real hand, with the same orientation. Asynchrony was achieved introducing a small temporal delay (0.5 s) between stroking the rubber hand and real hidden hand^11^ (Fig. 1).

The *Sight-Robotic_Touch-Real* condition was the synchronous version of the control condition, employing as fake hand a left robotic hand with a rubber cover matching the participant’s gender.

In all the *Touch-Real* conditions tactile stimulation was delivered by stroking with a paintbrush the real left hand of the participant.

In all the *Touch-Virtual* conditions tactile stimulation was delivered through two vibrators placed on the proximal and distal phalanx of the index finger (Fig. 1) which delivered a vibration matched in time, duration and spatial sequence (proximal to distal) to the visual stimulation.

The *Sight-Real_Touch-Real* condition was achieved by stroking the real, and visible, left hand of the participant. This condition was representative to what extent a subject consider embodied her/his real hand, filtered by the confounding factors that the experimental setup and circumstances can add to that estimation. Despite we are aware that the *Sight-Real_Touch-Real* condition is not a perceptual illusion, we thought helpful to add it to the study design because it allows to estimate the maximum values of the chosen measures of embodiment, being the visible hand really part of the tested subject’s body. These maximum values were employed to normalize the data of the other conditions.

The *Sight-Real_Touch-Virtual* condition was achieved by stimulating the hidden real left hand with vibrators, while showing to the subject their mirrored right hand that from their point of view seemed to be stroked with a paintbrush. None of the subjects declared that the seen hand was actually their contralateral one. 19 out of 26 subjects were tested for the *Sight-Real* conditions.

All the *Sight-Virtual* conditions were delivered through a HMD (Fig. 1) which displayed an ad hoc virtual environment. In particular, during the *Touch-Real* condition the experimenter delivered, with the help of a metronome, a brushstroke stimulation synchronous to the same movement on a virtual hand presented by the system (Fig. 1); in this case the system alerted the experimenter by providing a specific sound before the touch of the paintbrush in the virtual environment. Here, the experimenter was trained to deliver the touch stimulation after a fixed time-period from such sound. For the *Touch-Virtual* condition, the stimulation was directily provided by the vibrotactile array (see Supplementary Material for images of the setup). To ensure that in all conditions the visible hand was placed 15 cm medially to the real hand the VR controller was employed to track the position of the real hand in the VR environment. Before the beginning of the experiment, the experimenter positioned the controller over the participant’s hand placed still on platform and tracked the hand’s position. After such calibration,the participant was asked to not move his/her hand.

Seven volunteers of the group of participants could not participate to the *Sight-Real* conditions, because these conditions were inserted in a later stage of the experiment; however, for both sub- groups of volounteers, the experimental sessions were carried out in two different days, at least a week apart from one another. In case of the sub-group of participants that performed all seven conditions, four conditions were administered in the first session and three in the second one; in case of the other sub-group three conditions were administered in the first session and two in the second one. The order of the experimental conditions was randomized across the subjects and sessions.

### Embodiment measures

The strength of illusion in the different conditions was quantified by four state of art measures^11,31^: three outcomes of a self-evaluation questionnaire and the proprioceptive drift (PD).

#### Questionnaire

Each participant filled-in a questionnaire (one after each experimental condition). The questionnaire included a *statements list* first designed by Botvinick and Cohen^11^ and translated in Italian. A part of the statements measured the illusion level (Illusion statements) and another part measured the suggestibility of the participants (Control statements). The list of the statements were slightly adapted from the traditional statement list, considering the type of visible hand in the different conditions (*Virtual* vs. *Robotic*). In case of *Sight-Robotic* and *Sight-Virtual* conditions there were six control statements. In case of *Sight-Real* conditions, three of the control statements were not applicable (i.e. the ones relative to the appearance of the hands), so, they were not presented (Table 1). Participants were asked to rate the extent to which these statements did or did not apply to them by using a seven-point Likert scale (ranged from −3 and 3). The *RHI index*, defined as the difference between the mean of rates of the pooled illusion statements and of the control statements, was calculated for each condition and employed as the illusion measure in the following analyses^33^. In addition to the statements list, participants were asked to rate *vividness* and *prevalence* of self-attribution of the artificial hand^11,25,34^. The vividness was defined as how life-like and realistic the illusion was when it was experienced (rated from 0 to 10). The prevalence rating (from 0 to 100%) reflected the amount of time in which the illusion was experienced.

**Table 1:** List of statements for the different conditions.

Number	Condition*	Item
1	Common	It seemed as if I were feeling the tactile stimulation at the location where I saw the visible hand touched
2	Common	It seemed as though the stimulation I felt was caused by the touch on the visible hand
3	Common	I felt as if the visible hand was mine
4	Robotic-Virtual	I felt as if the position of my real hand was drifting towards the alien hand
	Real	I felt as if the position of my real hand was drifting towards the right side
5	Common	It seemed as if I had more than two hand or arm
6	Robotic-Virtual	It seemed as if the tactile stimulation I was feeling came from somewhere between my own hand and the visible one
	Real	—
7	Robotic	I felt as if my real hand were turning ‘rubbery’
	Virtual	I felt as if my real hand were turning ‘virtual’
	Real	—
8	Robotic-Virtual	It appeared as if the position of the visible hand was drifting towards my real hand
	Real	It appeared as if the position of the visible hand was drifting towards the left side
9	Robotic-Virtual	The alien hand began to resemble my own hand, in terms of shape, skin tone, freckles or some other visual features
	Real	—

*Condition: *Sight-Real*: Real, *Sight-Robotic*: Robotic, *Sight-Virtual*: Virtual.

#### Proprioceptive drift

It was reported that, after synchronous stimulation, the perceived location of participant’s hand shifts towards the rubber hand: this effect has been known as proprioceptive drift (PD). Such PD (as defined by Tsakiris and Haggard^31^) was calculated as the difference between the measurements of post- and pre-stimulation pointing task for each condition. During the pointing task measurement, the participants indicated with their right hand, while closing their eyes, the felt position of the tip of their left second digit. In all conditions, except for the *Sight-Real* one, the visible hand was set 15 cm medially to the stimulated hand, while in the *Sight-Real* conditions, real and visible hand matched. In order to make comparison across conditions, PD in the *Sight-Real* conditions was calculated as if the real hand was 15 cm away the visible hand, thus subtracting to 15 cm the distance between the post-stimulation pointing task measure and the actual position of the hand. In such way, a value of proprioceptive drift equal to 15 cm corresponds to correct identification of the position of the visible hand; whereas lower values indicate a discrepancy with the visible hand’s actual position.

### Embodiment measures analysis

Data from embodiment measures were collected and organized on the basis of the condition. The Shapiro-Wilk test (p > 0.05) was used to verify that the data were normally distributed.

To confirm that the recruited subjects were not suggestible or, in other words, that illusion statements would get a higher mean score than the control statements, a two tailed paired t-test, or signed rank test on basis of the data distribution, were employed^11,25,34,35^. Signed rank test was employed just for *Sight-Real_Touch-Real, Sight-Virtual_Touch-Real* and *Control* condition.

In order to confirm that each experimental condition was different from the typical no illusion control condition (asynchronous RHI), six pre-planned pairwise comparisons were performed on the extracted measures (i.e. RHI index, proprioceptive drift, and vividness and prevalence scores) by means of paired t-test or Wilcoxon signed-rank test when not normally-distributed, and Bonferroni corrected (degree of freedom of the analyses equal to 18 and 25 for Real and the other conditions, respectively). A p-value less than 0.05 was considered as statistically significant. Signed rank test was employed for the comparison of the *Control* condition with all the other ones for proprioceptive and vividness score and just for the comparison with *Sight-Real_Touch-Real* condition for RHI index and prevalence score data.

Once established that the illusion happened in all synchronous conditions, in order to remove any confounding common effect due to the experimental procedure, for all the collected measures and in each participant, the score of the asynchronous control condition was subtracted from the relative score of synchronous condition. The obtained outcomes were defined as *Δ scores*.

The Δ scores were analyzed with a linear mixed-effects model in case of RHI index and proprioceptive drift. Generalized linear mixed model was employed in case of vividness and prevalence scores. For all the analyses, participants were modelled as the random effects factor, and type of visual (*Sight:* Real vs. Robotic vs. Virtual) and tactile feedback (*Touch*: Real vs. Virtual) were modelled as the fixed effects factors. Therefore, the data were processed in an ANOVA-like analysis, resulting in a 3 × 2 model design. This has several advantages over the classic, repeated measures ANOVA approach: it allows to effectively fit large and unbalanced data sets (e.g. missing data of the *Sight-Real* conditions in our case) and requires less restrictive assumptions to run the analysis properly^36^.

Nine post-hoc pre-planned pairwise comparisons were performed on Δ scores by compairing all the pairs that maintain one factor-level (e.g. *Touch-Real*) in common and significance was Bonferroni-corrected accordingly because we have no interest in evaluating the embodiment effect by crossing the level between factors.

### Principal component analysis

A principal component analysis (PCA) was performed on the normalized Δ scores dataset in order to simplify the analysis and identify a single feature capable to resume the common informative content of the data. Dataset normalization was performed by using z-score transformation. Since PCA is a mean removal process, the offset, calculated as the principal component (PC) score obtained by a null Δ scores vector (i.e. no difference with *Control* condition), was added to the PCA outcomes. The more relevant offset-included principal components (i.e. the first PC of the Embodiment, 1PCE) were analyzed with an ANOVA-like linear mixed-effects model (fixed effects factors: *Sight* and *Touch*; random effects factor: participants).

The values of the 1PCE were employed to quantify the difference on illusion level for the different conditions, in particular between the condition of embodiment of the real limb (i.e. *Sight-Real_Touch-Real* condition) and the other ones.

The dimensionality reduction at the base of the PCA does not resume part of the data variance, thus further analyses based on the extracted 1PCE may not be completely adherent to the one extracted from raw data analyses. However, PCA is among the best options to resume all the collected data in a single value, making further analysis, and consequent findings, more simple and straightforward.

Nine post-hoc pre-planned pairwise comparisons were performed on 1PCE by compairing all the pairs that maintain one factor-level (e.g. *Touch-Real*) in common and significance was Bonferroni-corrected accordingly because we have no interest in evaluating the embodiment effect by crossing the level between factors.

A further statistical evaluation was performed in order to assess the effect of concordance between the level of virtualization of visual and somatosensory inputs. Concordance was considered when both the input modalities were physical (real or robotic) or both virtual, discordance when a modality was physical and the other virtual. The concordant conditions (*Sight-Real_Touch-Real*, *Sight-Robotic_Touch-Real*, *Sight-Virtual_Touch-Virtual*) were compared, by means of a paired t-test, to the discordant ones (*Sight-Real_Touch-Virtual*, *Sight-Robotic_Touch-Virtual*, *Sight-Virtual_Touch-Real*). Moreover, in order to exclude the strong effect on embodiment outcomes due to *Sight-Real_Touch-Real* conditions from the analysis, a two-way repeated measures ANOVA (factors: *Sight* and *Touch*) was also employed removing the *Sight-Real* conditions.

The surface of Fig. 4 represents the best fit surface of the blue dots, where each single dot represents a participant in each condition. The point for each subject was identified by three coordinates in a 3D space where z-value was the 1PCE, x-value was the marginal effect on the 1PCE of *Sight* pooling together all the levels of factor *Touch* and y-value was the marginal effect of *Touch* pooling together the level of factor *Sight*. The marginal effect is the effect on the dependent variable of one factor pooling together all the levels of the other factor (e.g. the marginal effect of *Sight-Real* condition for each subject was equal to the mean 1PCE value of *Sight-Real_Touch-Real* together with *Sight-Real_Touch-Virtual* conditions for that particular subject). The data were fitted on polynomial curves (maximum tested order equal to 5). Several factors were taken into account for the selection of fit curve. The selected curve, besides having a normal distribution of the residuals, has the best trade-off between the goodness of fit (i.e. coefficient of determination R^2^) and the polynomial order employed in curve model. In particular, the curve with a small number of polynomial coefficients (less than 15) and the highest value of goodness of fit was selected.

The datasets generated during and/or analysed during the current study are available from the corresponding author on reasonable request.

## Supplementary information

Supplemetary informations

Dataset 1
