# Supplementary material for: Different level of virtualization of sight and touch produces the uncanny valley of avatar’s hand embodiment
Source: Sci Rep. 2019 Dec 13;9:19030. doi: 10.1038/s41598-019-55478-z (PMC6911036; doi:10.1038/s41598-019-55478-z)
Supplement: Supplementary file 1 — Supplemetary informations [file 41598_2019_55478_MOESM1_ESM.pdf]

Different level of virtualization of sight and touch produces the uncanny valley of avatar's hand embodiment

M. D'Alonzo[a], A. Mioli[a], D. Formica[a], L. Vollero[a] and G. Di Pino[a*]

[a] *NeXT: Neurophysiology and Neuroengineering of Human-Technology Interaction Research Unit, Campus Bio-Medico University, via Alvaro del Portillo, 200, 00128, Rome, Italy*

SUPPLEMENTARY INFORMATION

Additional figures

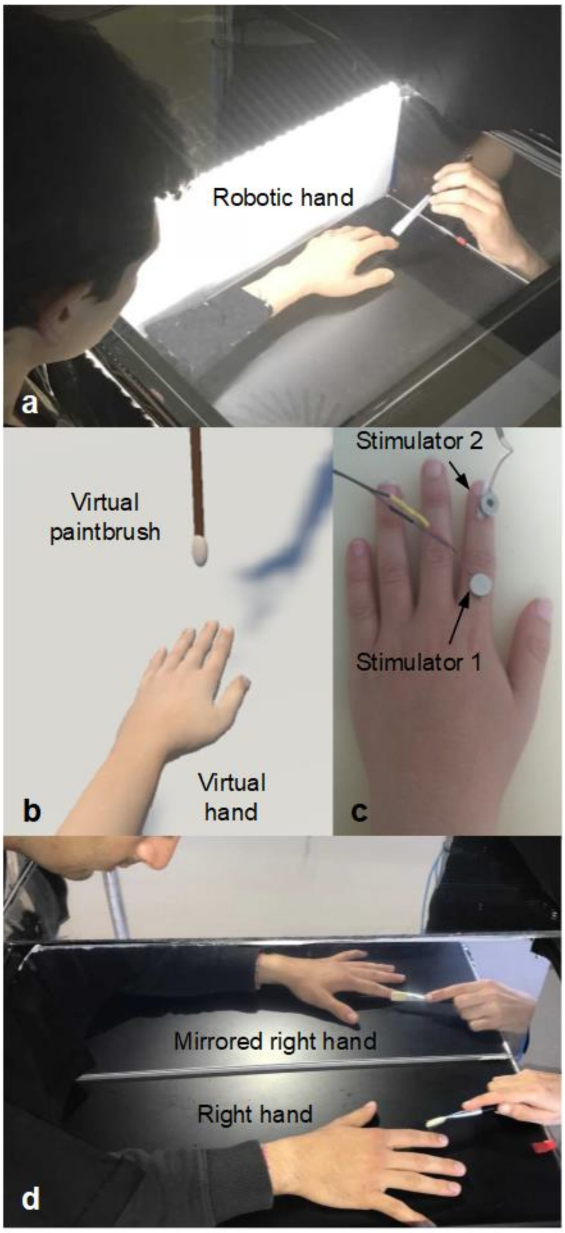

Fig. S1: Experimental set-up during the different experimental sessions. a) Experimental set-up during *Sight-Robotic* conditions. The informed consent for use of the image was signed by the participant. b) Screenshot of VR application as viewed through the HMD. c) Particular of the placement of stimulators on the hand of a participant during *Touch-Virtual* conditions. d) Set-up during *Sight-Real_Touch-Virtual* condition (during such condition the right hand was no visible to the participant by covering part of the platform frame). The informed consent for use of the image was signed by the participant.

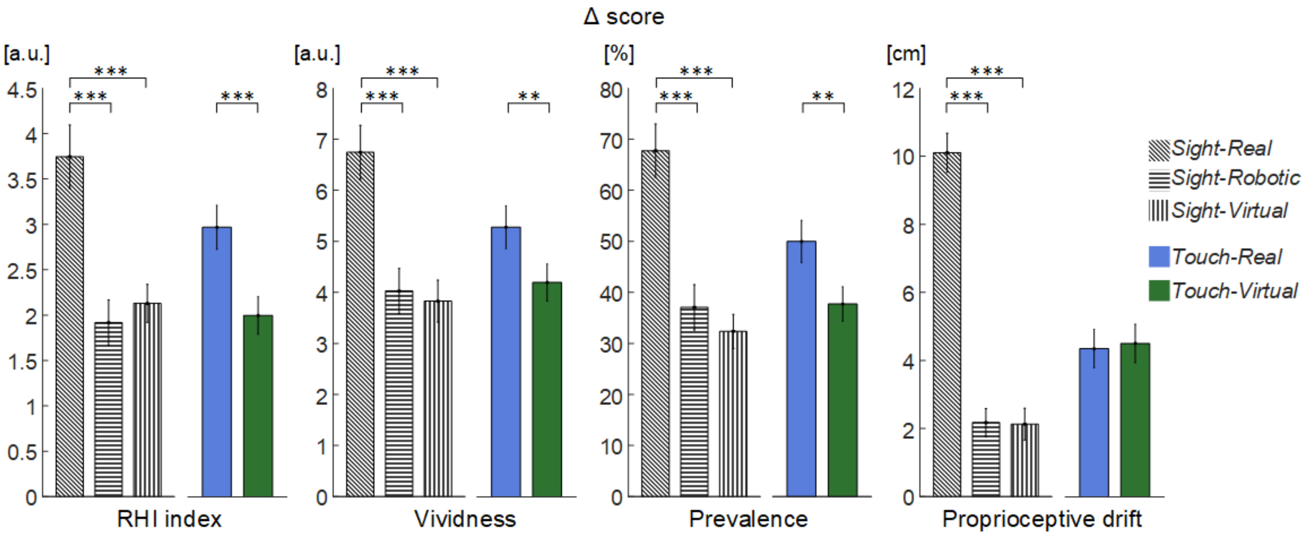

Fig. S2: Δ scores (mean ± standard error) of the RHI index (difference between the means of pooled illusion statements and control ones), vividness, prevalence rating and proprioceptive drift for different degree of virtualization. The graphs are separated for Sight and Touch factors. Different patterns of the bars were employed to differentiate the degree of Sight virtualization, whereas different colors differentiate the degree of Touch virtualization. ** indicates a p-value < 0.01; *** indicates a p-value < 0.001.

Analysis of embodiment measures excluding Real-Sight conditions

A two-way repeated measures ANOVA (factors: *Sight* and *Touch*) was also employed on the Δ scores of embodiment outcomes removing the *Sight-Real* conditions, in order to exclude from the analysis the strong effect due to these conditions. Considering each Δ score measure independently, an interaction has been observed for the outcomes of the *RHI index* (interaction: $F(1,25) = 5.2$, $p = 0.032$), *vividness* (interaction: $F(1,25)= 4.3$, $p = 0.049$) and *prevalence* of the illusion (interaction: $F(1,25) = 7.8$, $p < 0.010$); no effects were found for the *proprioceptive drift.*

PCA results

The analysis of correlation coefficients (ρ) among the illusion outcomes highlighted a high correlation between the prevalence and vividness ratings ($\rho = 0.85$, $p < 0.001$) and lower correlation values for the other tests between pair of outcomes (ρ values ranged between 0.35 and 0.45, $p < 0.001$). The four PCs explained the 61, 19, 16 and 4 % of variance of the dataset, respectively. The highest component loadings of PCA matrix relative to the first PC were associated to prevalence (0.58) and to vividness (0.55), with a lesser extent, to RHI index (0.43) and proprioceptive drift (0.42). The loadings for the second PC were positive for RHI index and proprioceptive drift (0.51 and 0.61, respectively), and negative for vividness and prevalence (-0.51 and -0.50, respectively), meaning a positive correlation for the first two measures and a negative one for the others with such PC; the loadings of the third PC for proprioceptive drift was positive (0.74) and, negative for RHI index (-0.67), the other loadings for such PC were in absolute value less than 0.05; the loading of fourth PC was positive for the prevalence (0.74) and, negative for vividness (-0.67) the other loadings for such PC were in absolute value less than 0.1.

Our analysis focused on the first PC that mainly describes the behavior of the data. Since the illusion outcomes describes different aspect of embodiment (i.e. intensity, continuance, illusion level with respect the suggestibility of the subject, and spatial update of sense of hand's position), a different variance of data distribution among conditions could indicate a different effect of the embodiment aspects; our interest was, hence, to evaluate whether the distribution of the outcomes was affected by the different conditions. For such reason, a correlation analysis between the first PC and the illusion outcomes for each conditions was performed, the obtained correlation values were compared to those ones calculated pooling all conditions together by using a Wilcoxon rank sum test. In general, the correlation coefficients calculated for each conditions were similar to those ones calculated on all data distribution ($p > 0.05$ for all four collected measures): The correlation coefficient of prevalence and

vividness with the first PC calculated on the global data were equal to 0.91 and 0.86, respectively; whereas, for the single condition, ranged from 0.81 (*Sight-Virtual_Touch-Virtual* condition) and 0.94 (*Sight-Robotic_Touch-Real*) and from 0.76 (*Sight-Real_Touch-Real*) and 0.91 (*Sight-Robotic_Touch-Real*), respectively. In case of the RHI index and proprioceptive drift the correlation coefficient calculated on the global data were equal to 0.66 and 0.65, respectively, the coefficient values for the single conditions were slighty lower: ranged between 0.42 (*Sight-Real_Touch-Virtual*) and 0.65 (*Sight-Robotic_Touch-Virtual*) and between 0.30 (*Sight-Real_Touch-Real*) and 0.52 (*Sight-Virtual_Touch-Real*), respectively. Globally, these results mean that the different aspects of embodiment had similar effect on data distribution for the other conditions.
